# Getting kids active by participating in sport and doing It more often: focusing on what matters

**DOI:** 10.1186/1479-5868-9-86

**Published:** 2012-07-12

**Authors:** Sandra Mandic, Enrique García Bengoechea, Emily Stevens, Sophia Leon de la Barra, Paula Skidmore

**Affiliations:** 1 School of Physical Education, University of Otago, PO Box 56, Dunedin, New Zealand; 2 Department of Kinesiology and Physical Education, McGill University, Montreal, Canada; 3 Department of Preventive and Social Medicine, University of Otago, Dunedin, New Zealand; 4 Department of Human Nutrition, University of Otago, Dunedin, New Zealand

**Keywords:** Adolescents, Sports, Physical activity, Sport management, Schools, Social determinants

## Abstract

**Background:**

Reduced time dedicated to physical education and free play in recent decades emphasizes the need to promote opportunities for sport participation in adolescents in order to increase physical activity levels. The purpose of this study was to examine the association of sociodemographic and biological characteristics, behavioural patterns, and school-related and sport-specific variables with time spent participating in sport.

**Methods:**

A total of 1837 secondary school students (age: 14.6 ± 1.2 years; 50.9 % boys) from 19 of 23 schools in the Otago Region (New Zealand) completed an online sport survey and Youth Physical Activity Questionnaire in 2009. Using multilevel modeling, we examined the association of individual-, school- and sport-related variables on sport participation and the amount of time spent in sports.

**Results:**

Higher rates of sport participation were associated with lower neighbourhood deprivation scores (OR (95%CI): 0.75 (0.49-1.14), 0.57 (0.38-0.86), 0.48 (0.28-0.81)), higher quintiles of physical activity (2.89 (2.10-3.96), 2.81 (1.68-4.70), 3.54 (2.24-5.57), 3.97 (1.99-7.95)), highest quintiles of screen time (1.58 (0.94-2.65), 1.99 (1.42-2.80), 2.17 (1.43-3.30), 1.88 (1.37-2.57)) and boys only school status (2.21 (1.57-3.10)). Greater amount of time spent in sports was associated with male gender (0.56 (0.43-0.74), lower neighbourhood deprivation scores (0.72 (0.59-0.93), 0.78 (0.58-1.04), 0.62 (0.39-1.00)), higher quintiles of physical activity (3.18 (2.29-4.41), 4.25 (2.91-6.20), 8.33 (5.58-12.44), 6.58 (4.07-10.64)), highest quintile of screen time (1.83 (1.31-2.56), greater availability of sports outside school (1.68 (1.22-2.32)), better sport management (2.57 (1.63-4.07)) and provision of sport courts at school (0.57 (0.40-0.81)). Conversely, obesity was associated with less time spent participating in sport (0.50 (0.31-0.80)).

**Conclusion:**

Results support the use of sport participation as an effective strategy to increase physical activity levels and identify target groups and areas for interventions, program design and policy development. Interventions should focus on improving accessibility to sport programs for all adolescents, providing adequate sport grounds at school, and promoting good sport management practices. Programs and policies encouraging sport participation should address in particular the needs of adolescents living in deprived neighborhoods, those attending coeducational and girls-only schools, and those who are obese.

## Background

Lack of physical activity (PA) and sedentary lifestyle in school-age children is a global health problem in both developed [1] and developing countries. [2] PA during childhood and adolescence has multiple health benefits. Therefore, opportunities to engage youth in PA such as sports, physical education classes, active transport and unstructured physical activities, are critical in encouraging healthy lifestyles in adolescents. With less time dedicated to physical education and free play in the recent decade, there is a greater need to promote opportunities for structured and unstructured PA for youth. [3] In addition, sports also provide important opportunities for students to contribute to the school community, which may cultivate an increased commitment to the school and school values [4].

Recent New Zealand data showed that 60% of boys and 50% of girls participate in sports outside of school time. [5] Participation in sports is associated with higher levels of PA in adolescents[6], contributes to 60% of daily moderate-to-vigorous PA in youth [7] and is one of the best predictors of PA in early adulthood. [8] Since approximately half of the youth sport practice time is spent in either sedentary or light-intensity activities [6,9], the actual percentage of youth meeting PA guidelines through sport participation most likely is overestimated.

Previous studies have examined factors associated with sport participation in adolescence using individual and school level variables including gender, socioeconomic status, weight status, self-efficacy for PA, andenjoyment of PA and physical education, and school climate for PA.[10-12] Extending previous research, our study investigates relevant sociodemographic and biological characteristics of students, behavioural patterns, and school-related variables as potential correlates of sport participation and time spent participating in sport. In addition, we designed a survey and incorporated sport specific variables that have not been addressed in the past to examine their potential contribution to time spent participating in sport (such as availability of sports, suitability of competitions and coaches, quality of sport management and schools provisions and resources for sports). Study variables were chosen based on the physical activity and sport literature and on their relevance to practitioners, as determined in consultation with the Otago Secondary School Sports Association. Previous research found that school-age students were more likely to engage in PA when schools provided adequate space, sport grounds and adult supervision. [13] These factors may impact significantly students experience in sport, their motivation and opportunities for participation.

The purpose of this study was two-fold: 1) to examine individual- and school-related factors that are associated with sport participation in secondary school students; and 2) to examine individual-, school- and sport-related factors that influence amount of time spent in sports among sport participants. Exploring relevant factors associated with adolescents’ participation in sport is important to take into account, since the greatest public health benefit is likely to occur from encouraging sedentary individuals to engage in physical activity. [14]. In addition, to maximize public health benefit along with other potential developmental and educational benefits derived from sport participation, it becomes critical to understand the influential factors, including sport-specific factors, associated with the amount of time spent in sport.

## Methods

### Study design

The Otago School Students Lifestyle Survey was conducted in 19 out of 23 secondary schools in the province of Otago, New Zealand, between October and December 2009. Cluster sampling was undertaken using schools as sample units. Purposive sampling was used with random classes from each school year invited to take part. A total of 2408 students from Years 9 to 13 were invited to take part in the web-based questionnaire. Students were given study information packages with separate information sheets and consent forms for student and parents. Students were required to sign a consent form to participate, while parents were only required to provide opt-out consent if they did not wish their child to participate.

The anonymous questionnaire was delivered and completed during classroom time. The questionnaire package included questions on demographics, PA, sedentary behaviour, sport participation, dietary habits, transportation habits and youth space preferences. Questionnaire items and data collection methods were pre-tested for comprehension and appropriateness by students from the target age group, school staff and experienced researchers. The online questionnaire was also pilot-tested to ensure that it could be completed within one class period and revisions were made to the questionnaire before data collection. The study was approved by the University of Otago Ethics Committee.

## Correlates and outcome measures

### Student characteristics and school variables

Basic demographic data collected included self-reported age, gender, and ethnicity. Neighbourhood socioeconomic status was assessed using the New Zealand Deprivation Index Score, which is derived from residential address and provides a measure of area level deprivation. [15] The deprivation index is an ordinal scale ranging from 1 (least deprived) to 10 (most deprived) and was used as a surrogate for students’ socioeconomic status. For the purpose of the analysis, four categories were created: lowest (1–2), middle-low (3–5), middle-high (6–8) and highest (9–10) deprivation score. Weight status was based on body mass index which was calculated from self-reported heights and weights. Participants were categorized as normal, overweight or obese using the cut-off points for body mass index based on international data [16].

Place of residence was determined based on school location in Dunedin city area (urban schools, n = 10) or greater Otago area (rural schools, n = 9). Educational status of the school included 3 categories: co-educational (n = 13), boys only (n = 3) and girls only schools (n = 3). Socioeconomic status of the school was based on school decile ranging from 1 (most deprived) to 10 (least deprived). School decile is determined by the proportion of students at the school with low socioeconomic status as defined by the student’s home address. Decile 1 contains the 10% of schools with the highest proportion of students from low socio-economic communities, while Decile 10 contains the lowest proportion of those students.

### Sport questionnaire

The sport questionnaire was developed in collaboration with Otago Secondary School Sports Association. The question “Are you involved in any sport or do you belong to any sport team?” was taken directly from New Zealand Youth ’07 Survey. [5] Students were asked the question “How many hours per week do you spend in sport in school or sport out of school?” with the following response options: “<1 hour per week”; “1-2 hours per week”; “3-5 hours per week”, or “≥6 hours per week”. Due to a small number of participants, we combined categories “<1 hour per week” and “1-2 hours per week” into “Up to 2 hours per week”.

Sport participants were asked to select up to 5 sports they participate in. For each sport, students were asked about availability, competitions, coaching, and management. A school sport resources score was created as a sum of individual responses to items related to school provisions and resources for sport ranging from 0 (low support) to 8 (high support). Students who did not participate in sports were asked about barriers that prevent them from participating in sports and were asked for suggestions for improving school sports. Students’ responses were analysed inductively searching for emerging patterns. The questions related to sport availability, competition, coaching and school provisions for sports were newly developed and pretested in a pilot study on 30 students in 1 school undertaken in September 2009.

### Physical activity and screen time

Self-reported PA (sports and other activities outside school, physical education classes, active transport) and screen time (playing computer games, using computer/internet, watching TV) in the previous week were assessed using a validated Youth Physical Activity Questionnaire for children. [17] Metabolic equivalents (METs) for each activity were determined from the compendium of PA for youth.[18] Thresholds values of 4 METs for moderate and 7 METs for vigorous PA were used [19]. PA data were truncated to 120 minutes per session to be consistent with objectively measured PA in adolescents. [20,21] Current threshold values of at least 60 minutes of moderate-to-vigorous PA per day and up to 2 hours of screen time per day were used to identify students meeting guidelines for each behaviour. [22] In addition, quintiles of PA and screen time were created for further analysis.

### Data analysis

We calculated descriptive statistics for all student demographics and school-level variables. To identify correlates of sport participation versus no participation, we conducted a multilevel logistic regression, using school as a cluster variable. This accounts for potential random effects in the dependent variable associated with clustering of the students within the schools. In this analysis, we examined univariate correlates of sport participation both at individual (age, gender, ethnicity, students’ socioeconomic status, weight status, PA and screen time) and the school level (school decile, school setting (urban versus rural) and school educational status). Significant univariate correlates were entered into a multivariate model.

Among sport participants, we examined factors associated with greater time spent participating in sport using a multilevel ordinal logistic regression, with school as a cluster variable. The Brant test indicated that the proportional odds assumption was not violated; therefore, it is appropriate and valid to use ordinal logistic regression for this model. In addition to individual and school level correlates examined in the logistic regression model, we also included variables related to sport management and school support for sports. These questions were only answered by sport participants. We followed the same approach by first examining correlates at the univariate level, and then entering significant univariate correlates into a multivariate model. Data were analysed using Stata Version IC11 (Stata Corp., College Station, TX, USA).

## Results

### Final study sample and descriptive statistics

Twelve students refused to participate and 311 were absent from the class. A total of 2085 students took part in this questionnaire. We excluded from data analysis 94 participants with invalid questionnaires, 94 participants with incomplete sport questionnaire data and 60 participants who did not provide details on sport participation. Therefore, a total of 1837 students were included in this analysis. Mean age of participants was 14.6 ± 1.2 years with a similar percentage of boys and girls (Table 1). Most participants reported New Zealand European origin, were of normal weight, lived in less deprived neighbourhoods, and lived in an urban area. Half of the participants attended co-educational schools. Among all schools in the Otago region, school decile indicated moderate to low deprivation (school decile ranging from 4 to 10).

**Table 1:** Student characteristics and school variables

	**Total**
	**n = 1837**
Age (years) (mean ± SD)	14.6 ± 1.2
Gender [n(%)]	
Boys	935 (50.9)
Girls	902 (49.1)
Ethnicity [n(%)]	
NZ European	1435 (78.1)
Maori	193 (10.5)
Other	209 (11.4)
NZ Deprivation score [n(%)]	
Low	489 (26.6)
Middle-low	691 (37.6)
Middle-high	479 (26.1)
High	112 (6.1)
BMI z-scores [n(%)]	
Under/normal weight	1230 (67.0)
Overweight	279 (15.2)
Obese	107 (5.8)
Place of residence [n(%)]	
Urban	1305 (71.0)
Rural	532 (29.0)
Co-ed school status [n(%)]	
Co-ed	976 (53.1)
Boys only	450 (24.5)
Girls only	411 (22.4)
School decile* (mean ± SD)	7.38 ± 1.87

*Range 1–10; higher score represents lower deprivation.

**Figure 1:**
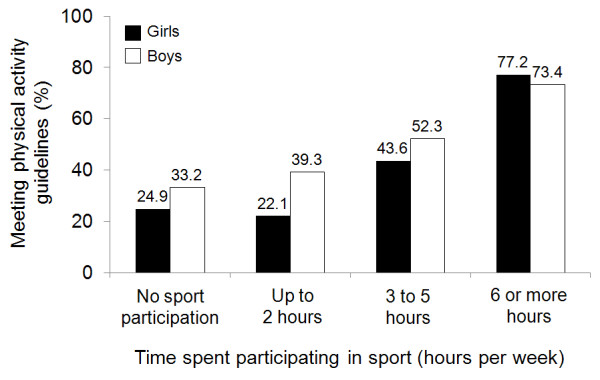
Percentage of boys and girls meeting physical activity guidelines among non-participants and across different categories of time spent in sport for sport participants.

A total of 1291 students (70%) participated in organized sports. The number of boys who participated was slightly higher than the number of girls (72.3% vs. 68.2%, p = 0.054). Sports participation was highest among adolescents from the least deprived neighbourhoods (77.5%) compared to students from the most deprived neighbourhoods (62.5%).The majority of sports were available at school and outside of school (Table 2). Most students perceived that sport competitions were suitable, coaches were available and suitable for the level of competition, and teams/sports were well managed. The majority of students playing sports perceived that their schools had sufficient sports grounds and the right sports gear, provided good organisation of sport at the school, acknowledged sport results and offered adequate opportunities for both best and social players (Table 2). Students also felt they had sufficient input regarding the organisation of sport in their school. The composite score for school sport and resources reflected positive perceptions of school support for sports. Among all study participants, only 33% of students reported spending ≥6 hours per week participating in sports. More time spent participating in sports was associated with an increased percentage of students meeting minimal PA guidelines (considering all types of PA) (no sport: 28.8%; up to 2 hours: 29.3%; 3 to 5 hours: 47.6%; 6 or more hours: 74.9%; p < 0.001). Gender-specific data are presented in Figure 1.

**Table 2:** Sport questionnaire items with descriptive statistics

	**Yes response**	
**Sport participants**	**(n = 1283)**	
Availability* (mean ± SD)		
Is your sport available at school? (%)	79.3 ± 31.9	
Is your sport available outside of school? (%)	85.4 ± 28.2	
Sport management* (mean ± SD)		
Are the [sport] competitions/games/events you compete in suitable for your level of performance? (%)	93.8 ± 17.4	
Do you or your team have a coach? (%)	77.8 ± 30.8	
Is your coach suitable for the level of competition? (%)	94.9 ± 18.5	
Is your sport/team well managed? (%)	83.4 ± 28.8	
School sport provisions and resources [n(%)]		
My school has enough sport grounds and courts	1098 (85.1)	
My school provides the right sport gear	1126 (87.2)	
Sport is well organized at my school	1093 (84.7)	
Everyone can participate in sports in my school	1184 (91.7)	
My school offers good opportunities for best players	1157 (89.6)	
My school offers good opportunities for social players	1083 (83.9)	
Students can influence sport organization	1117 (86.5)	
Sport results and successes are acknowledged in my school	1203 (93.2)	
School support resource score (range: 0–8)	7.3 ± 1.22	
**Students who do not participate in sports** (n = 546)		
**Barriers** [n(%)]	**At school**	**Outside school**
It costs too much	93 (17.0)	96 (17.6)
I'm not good enough at sports	180 (33.0)	167 (30.6)
I'm not interested	281 (51.5)	247 (42.2)
It takes too much time	167 (30.6)	157 (28.8)
None of my friends are in sports	127 (23.3)	114 (20.9)
The sports I'm interested in aren't available	147 (26.9)	77 (14.1)
Can't get there	94 (17.2)	107 (19.6)
I would feel shy, nervous or embarrassed	133 (24.4)	105 (19.2)
I have other responsibilities	144 (26.4)	117 (21.4)
My parents would not let me	26 (4.8)	31 (5.7)
I don't know the reason	84 (15.4)	91 (16.7)
Other	71 (13.0)	61 (11.2)

*Note: Percentages represent averages for all sports that students participated in.

### Correlates of sport participation

In univariate logistic regression analyses, significant correlates of sport participation were gender, age, student’s socioeconomic status, weight status, PA, screen time, and educational status of the school. In a multivariate model, significant correlates were student’s socioeconomic status, PA, screen time, and educational status of the school (Table 3). Compared to students in the highest socioeconomic category, each lower category of socioeconomic status was associated with lower odds of sport participation (p < 0.05). The reverse trend was observed for PA. Compared to the lowest PA quintile, each higher quintile was associated with increased odds of sport participation (p < 0.001). Higher quintiles of screen time were associated with sport participation (p < 0.01). Finally, students in boys only schools were more likely to participate in sports compared to students from co-educational schools (p < 0.001).

**Table 3:** Multivariate correlates of sport participation

		**OR**	**95% CI**	**SE**	**p-value**
		**n = 1837**			
Age		0.93	0.84-1.03	0.05	0.176
Gender		1.18	0.86-1.63	0.18	0.289
NZ Deprivation score					
	Low	1.00			
	Middle-low	0.75	0.49-1.14	0.15	0.166
	Middle-high	0.57	0.38-0.86	0.11	0.010
	High	0.48	0.28-0.81	0.12	0.008
Physical activity quintiles					
	<60 min/day	1.00			
	60-89 min/day	2.89	2.10-3.96	0.44	<0.001
	90-119 min/day	2.81	1.68-4.70	0.69	0.001
	120-179 min/day	3.54	2.24-5.57	0.77	<0.001
	≥180 min/day	3.97	1.99-7.95	1.31	0.001
Screen time quintiles					
	≤2 hours/day	1.00			
	2.1-2.9 hours/day	1.58	0.94-2.65	0.39	0.081
	3.0-3.9 hours/day	1.99	1.42-2.80	0.32	<0.001
	4.0-4.9 hours/day	2.17	1.43-3.30	0.43	0.001
	≥5.0 hours/day	1.88	1.37-2.57	0.28	0.001
Co-ed school status					
	Co-ed	1.00			
	Boys only	2.21	1.57-3.10	0.36	<0.001
	Girls only	1.12	0.79-1.59	0.19	0.518

*Table with univariate results is available from the first author.

### Correlates of time spent participating in sport

In univariate ordinal regressions, significant correlates of time spent participating in sport were gender, age, student’s socioeconomic status, weight status, PA, screen time, availability of sports outside school, quality of sport management, sufficient sport grounds at school, sport results acknowledgment at school, and educational status of the school. Significant multivariate correlates were gender, student’s socioeconomic status, weight status, PA, screen time, availability of sports outside of school, quality of sport management, and provision of sport grounds at school (Table 4). Among sport participants, girls spent less time participating in sports compared to boys (p < 0.001). In terms of socioeconomic status, students in the middle-high (p < 0.01) and lowest categories (p < 0.01) reported less amount of time spent participating in sports compared to the highest category. Obese students reported less time spent in sports compared to their normal/underweight counterparts (p < 0.001). Time spent participating in sport showed a trend to increase with each higher quintile of PA (p < 0.001) and was highest for the highest quintile of screen time (p < 0.001). Availability of sports outside school was associated with more time participating in sports (p = 0.001). Similarly, good management of sports (p < 0.001) and provision of sufficient sport grounds at school (p = 0.001) increased the odds of students spending more time participating in sports.

**Table 4:** Multivariate correlates of time spent participating in sport

		**OR**	**95 % CI**	**SE**	**p-value**
		**n = 1837**			
Age		0.95	0.85-1.05	0.05	0.282
Gender		0.56	0.43-0.74	0.08	<0.001
NZ Deprivation score					
	Low	1.00			
	Middle-low	0.74	0.59-0.93	0.09	0.009
	Middle-high	0.78	0.58-1.04	0.12	0.093
	High	0.62	0.39-1.00	0.15	0.048
Weight status					
	Under/normal weight	1.00			
	Overweight	1.00	0.70-1.42	0.18	0.999
	Obese	0.50	0.31-0.80	0.12	0.004
Physical activity quintiles					
	<60 min/day	1.00			
	60-89 min/day	3.18	2.29-4.41	0.53	<0.001
	90-119 min/day	4.25	2.91-6.20	0.82	<0.001
	120-179 min/day	8.33	5.58-12.44	1.71	<0.001
	≥180 min/day	6.58	4.07-10.65	1.62	<0.001
Screen time quintiles					
	≤2 hours/day	1.00			
	2.1-2.9 hours/day	1.09	0.72-1.64	0.23	0.689
	3.0-3.9 hours/day	1.17	0.86-1.59	0.18	0.322
	4.0-4.9 hours/day	1.33	0.94-1.88	0.23	0.110
	≥5.0 hours/day	1.83	1.31-2.56	0.31	<0.001
Availability of sport outside school		1.68	1.22-2.32	0.28	0.001
Quality of sport management		2.57	1.63-4.07	0.60	<0.001
Sufficient sport grounds and courts at school		0.57	0.40-0.81	0.10	0.001

*Table with univariate results is available from the first author.

### Barriers and suggestions for improvement

The main barriers to sport participation both at school and outside school were lack of interest in sports followed by low perceived competence, lack of time and other responsibilities (Table 2). Lack of available sports and self-presentational concerns were greater barriers for sport participation at school compared to outside school. Among 546 students who did not participate in organised sport, the most common suggestions for improvement in school sports included offering a wider range of sports (15.0%), making sport more fun and less competitive (8.8%), encouraging students to play sports (5.3%), organizing sports during school time (5.3%), involving friends, family and teachers (4.9%), giving awards or paying students (4.2%), making sports affordable or free (4.2%), promoting available sports (2.9%), and providing transportation or easier access to sports (2.6%). Other suggestions, mentioned less frequently, included offering sports lessons, allowing students to choose which games to play, providing more equipment and better coaches, changing the sport schedule, reducing bullying on sports teams, having less homework, offering traveling opportunities, supplying better uniforms, and playing music in the gym.

## Discussion

The results of the present study support the use of sport participation as an effective strategy to increase PA levels. Youth engages in PA through sports, active transport, physical education classes and unstructured PA. Sport participation is a significant component of daily energy expenditure in youth. [7] Similar to findings from United States [23], Canada [24] and New Zealand [5], 70% of secondary school students in Otago participate in sports**.** Even though sport participation may not protect against a decline in PA levels during adolescence [25], it is one of the best predictors of PA in early adulthood [8].

Adolescents are more likely to engage in PA when schools provided adequate space, sport grounds and adult supervision. [13] Secondary schools in the Otago region provide excellent support for sports. Availability of sports, adequate coaching and competitions, school support and multiple opportunities for participation most likely contribute to high sport participation rates in this study. Quality of sport management, availability of sports outside school and provision of sport grounds at school were the only modifiable dimensions which predicted more time spent participating in sport. Previous research shows that fun, success, variety, freedom, family participation, peer support and enthusiastic leadership are important for encouraging youth participation in organized sport. [3] Based on suggestions of students not participating in sport in the present study, other areas for school-based interventions include offering a wider range of sports at school, making sport more fun and less competitive, involving friends, family and teachers, and encouraging students to participate. Therefore, programs, interventions and policies designed for promoting PA through sport participation should focus on providing a well-organized sport environment with a wide range of competitive and non-competitive options, making sports available both at school and outside school, providing adequate space and facilities, and offering opportunities for the involvement of friends and family members. This way, promotional efforts can help address perceived barriers to participation identified in this study such as lack of interest and time, and low perceived competence for sports. These efforts should focus preferentially on obese adolescents, students attending co-educational and girls only schools, and, particularly, students living in more deprived neighbourhoods.

Previous studies have found that boys report spending more time in sport participation than girls[23,26]. In the present study, gender differences in sport participation rates were small (72% versus 68%) and of borderline statistical significance. However, boys spent more time participating in sports and significantly more boys were meeting minimal physical guidelines solely through sport participation (39% versus 27%). Several studies reported a greater total daily energy expenditure in youth sports[7] and higher levels of activity during a sport practice[9] in boys versus girls. These findings are consistent with higher PA levels reported by boys in the present study. In addition, educational status of the school was one of the significant multivariate correlates of sport participation in our study. Students in boys only schools were 2 times more likely to participate in sports compared to students from co-educational schools, suggesting the influence of the school culture regarding gender appropriateness of sport participation. One possible explanation for this finding is that boys only schools may be more likely to endorse and promote sport participation than co-educational and girls-only school as a result of gender stereotypes (e.g., prevailing media images) suggesting that sport participation is more appropriate and beneficial for males. Sport participation in girls-only and co-education schools could be promoted by fostering family, peer and teacher involvement and support, in particular those of females, who may act as role models; offering a variety of fun and non-competitive activities, and providing a wider range of sports, some of which may appeal more to girls. Participation could also be promoted by discussing with the students prevailing gender stereotypes about sport participation.

An alarming finding in our study was that lower socioeconomic status, as measured by neighbourhood deprivation score, emerged as a consistent and strong correlate of both participation in sport and time spent in sport. In fact, each lower category of socioeconomic status was associated with lower odds of sport participation. This confirms in a sample from New Zealand that inequities in availability of organized sport programs may also exist in schools and communities based on socioeconomic gradients, corroborating previous findings from Canada and Australia. [11,27,28] In addition, the gradient we observed may reflect lower propensity of lower socioeconomic status individuals to sign up for and persist in a variety of health promotion programs.[29] Improving availability and affordability of developmentally appropriate sport programs for students with lower socioeconomic status, offering a variety of programming choices, providing adequate school grounds for participation, making sure support and encouragement are offered on an ongoing basis, and ensuring opportunities for participants to interact with positive role models, may help these students overcome some of the barriers they face.

Obese adolescents in this study spent fewer hours participating in sport than their normal weight/underweight peers, which is consistent overall with research from Australia [28] and Canada [11]. These adolescents may struggle with issues of body image, and in some cases their skill and fitness levels may not be on par with those of their peers. Consequently, the motivation of those considering participation in sport may be affected negatively. This finding also indicates that obese adolescents already participating in sport may not enjoy their experience as much and be less inclined to persist. Careful planning of the sport environment offered to obese adolescents remains crucial. For example, practices and competitions can be structured in a manner that leads obese adolescents to progressively develop their skills and improve their fitness levels. Likewise, creating an inclusive environment where learning, improvement, effort, and enjoyment are prioritized and reinforced as main goals for participation may contribute to a positive and rewarding experience in sport for obese adolescents as well.

Finally, the findings may seem counterintuitive that both higher rates of sport participation and more time spent participating in sport were associated only with the highest quintile of screen time in our sample; these findings parallel research that indicates the relationship between screen time and physical activity participation is complex. [30] For example, students who participate often in sports may also spend considerable amounts of time in front of the television watching their favorite sports. These students may benefit from being reminded of the negative health consequences of too much time spent sitting in front of the screen, regardless of how physically active they are.

In this study, only one third of students reported meeting minimal PA guidelines solely through participation in organized sports. Taking into account that many sports and related practice drills do not require or provide sufficient PA and energy expenditure to achieve measurable health and fitness advantages (especially for novices) and often involve too much standing or walking around [9,31], this percentage of students meeting PA through sport participation is likely to be overestimated. Studies of youth sport practices found that approximately half of the practice time (43% to 54%) is spent in sedentary or light-intensity activities. [6,9] The situation may be further exacerbated by limited resources and space for sport practices, inappropriate coach-to-child ratios, and personal priorities of coaches and administrators resulting in less than adequate amount and intensity of PA associated with sport participation.[31]

Sport participants reported a greater weekly amount of moderate-to-vigorous PA compared to their peers. [32] In our study, both sport participation and the time spent in sports were associated with higher levels of PA. The percentage of students meeting PA guidelines increased significantly from 29% of students who participate in sports up to 2 hours per week to 75% of students who participate for 6 or more hours per week. Therefore, although sport participation represents an important opportunity for increasing PA in youth, this alone may not be sufficient for meeting PA recommendations in most adolescents. To increase overall exercise intensity and energy expenditure and maximize activity and playing time, sports program organizers need to plan to establish teams and groups with small number of students per coach, court and field. [31] Coaches should ensure that activities during sport practices are structured to maximize PA intensity and involvement for all participants. In addition to organized sports, adolescents need to be encouraged to adopt an active lifestyle, incorporate PA in their everyday life such as active transport, and engage in physical activities outside of organized sport.

Study limitations include lack of random sampling of students, which was not feasible in the present study. However, we recruited 19 out of 23 schools in the Otago region of New Zealand. The non-participating schools were high decile and therefore students with lower socioeconomic status were not undersampled in the present study. In addition, findings are based on self-reported data, which may have introduced response biases such as acquiescence and social desirability biases. The careful design of the survey questions, along with pre-testing and pilot testing, as explained in the Methods section, also contributed to prevent or minimize biases. The cross-sectional design precludes comparing data across different seasons and the possibility of making causal inferences. Future studies should consider incorporating longitudinal designs.

In summary, both sport participation and the time spent participating in sports are associated with higher levels of PA in adolescents. Interventions, programs and policies should address the needs of adolescents living in more deprived neighbourhoods and those who are obese, while being sensitive to the culture of the school regarding stererotypes about what is gender appropriate. Possibilities for intervention include increasing availability of sports, improving the quality of sport management, providing sport grounds at school, offering a wide range of enjoyable competitive and non-competitive options, and fostering interaction with family, peers, and teachers. Encouraging adolescents to participate in sports and providing them with opportunities to do so is an effective strategy to increase overall PA in youth. Adolescents who do not enjoy participating in organized sports should be encouraged to engage in other forms of PA such as unstructured activities, active transport and physical education classes.

## Abbreviations

METs, Metabolic equivalents; PA, Physical activity.

## Competing interests

The authors declare that they have no competing interests.

## Authors’ contributions

SM and PS were the principal investigators for this overall project, contributed to the conception and design of the project and monitored questionnaire design and data collection process. SM also contributed to data analysis and interpretation for this study, drafted and revised the manuscript. PS also revised the draft manuscript. EBG contributed to conceptualization of this particular study, data analysis and interpretation and manuscript writing and revisions. ES assisted with data collection, management and analysis and manuscript revisions. SLB assisted with data analysis and interpretation and revised the draft manuscript. All authors read and approved the final manuscript.
